# fNIRS-VFT is a complementary tool to ^18^F-FDG PET in presurgical evaluation of drug-resistant epilepsy: an exploratory study

**DOI:** 10.1186/s42494-026-00282-2

**Published:** 2026-10-26

**Authors:** Shuting Hong, Huiqin Xiao, Chengjun Li, Xuemiao Lai, Zhen Mei, Xiaofen Huang, Yu Tian, Lin Lin, Yue Pang, Zinan Luo, Ying Zhang, Lingbo Zeng, Feng Wang, Yuanxiang Lin

**Affiliations:** 1 Department of Neurosurgery, the First Affiliated Hospital, Fujian Medical University, Fuzhou, 350005 China; 2 Neurosurgery Research Institute, The First Affiliated Hospital, Fujian Medical University, Fuzhou, 350005 China; 3 Department of Neurosurgery, Binhai Campus of the First Affiliated Hospital, National Regional Medical Center, Fujian Medical University, Fuzhou, 350212 China; 4 Department of Nursing, the First Affiliated Hospital, Fujian Medical University, Fuzhou, 350005 China; 5 Department of Nuclear Medicine, the First Affiliated Hospital, Fujian Medical University, Fuzhou, 350005 China; 6 Fujian Provincial Institutes of Brain Disorders and Brain Sciences, First Affiliated Hospital, Fujian Medical University, Fuzhou, 350005 China

**Keywords:** Drug-resistant epilepsy, Surgery, ^18^F-FDG-PET, fNIRS, Epileptogenic zone localization, Verbal memory

## Abstract

**Background:**

Accurate presurgical localization of the epileptogenic zone (EZ) and preservation of postoperative cognitive function are two critical but often competing goals in epilepsy surgery for drug-resistant epilepsy (DRE). This study aimed to (1) evaluate the complementary value of functional near-infrared spectroscopy during a verbal fluency task (fNIRS-VFT) alongside interictal ^18^F-FDG PET for presurgical EZ localization; and (2) explore whether task-based fNIRS functional connectivity is associated with postoperative verbal memory decline.

**Methods:**

We retrospectively included patients with DRE who underwent epilepsy surgery between December 2022 and December 2023 and completed both interictal PET and fNIRS-VFT assessments. All analyses in this study were descriptive, and no robust inferential statistical testing was performed. For the localization outcome, concordance between PET hypometabolism and fNIRS-VFT hypoactivation was analyzed in patients who achieved seizure freedom two years after surgery. For the memory outcome, post- vs. pre-operation change of task-based fNIRS functional connectivity was compared between patients with and without clinically significant postoperative verbal memory decline (≥ 4 point reduction on Auditory Verbal Learning Test delayed recall).

**Results:**

Twenty-eight patients were included in this study. Among 20 patients achieving seizure freedom, PET demonstrated concordant lateralization with the EZ in 14 cases (70%), including 11 with accurate localization; fNIRS-VFT showed concordant lateralization in 9 patients (45%), with 4 accurate localizations including one case in whom PET failed to lateralize the EZ, indicating the complementary value of fNIRS-VFT. Among 19 patients with complete memory data, four showed significant postoperative decline. These patients exhibited more pronounced alterations in task-related functional connectivity, particularly involving frontopolar, dorsolateral prefrontal, premotor, and temporal regions.

**Conclusions:**

In this exploratory, descriptive analysis, interictal ^18^F-FDG PET showed higher concordance with lateralization of surgical areas compared with fNIRS-VFT among patients with DRE. Nevertheless, fNIRS may provide complementary information for cases with inconclusive PET findings. Additionally, changes of task-based fNIRS connectivity may be correlated with postoperative verbal memory alterations. These preliminary findings suggest that fNIRS-VFT may provide complementary information in selected cases, which warrants validation in larger prospective cohorts.

**Supplementary Information:**

The online version contains supplementary material available at https://doi.org/10.1186/s42494-026-00282-2.

## Background

Drug-resistant epilepsy (DRE) refers to a form of epilepsy in which seizures remain uncontrolled after trying at least two tolerated, appropriately chosen antiseizure medications at effective doses [1]. In addition to recurrent seizures, patients often experience substantial cognitive, emotional, and social impairments [2, 3]. Surgical resection of the epileptogenic zone (EZ) is an effective treatment option for patients with DRE, significantly reducing seizure frequency and improving the quality of life of patients [4, 5]. However, the success of epilepsy surgery critically depends on accurate presurgical localization of the EZ [6].

^18^F-fluorodeoxyglucose positron emission tomography (^18^F-FDG-PET) has become an important tool for presurgical imaging in DRE [7], assisting in the identification of EZ by detecting interictal hypometabolism and providing prognosis for surgical outcomes [8, 9]. Nevertheless, PET cannot be routinely used for ictal imaging, and it is also limited by radiation exposure, high cost, and restricted use in populations such as pregnant or breastfeeding women and young children.

Functional near-infrared spectroscopy (fNIRS) is a noninvasive, portable, and radiation-free neuroimaging technique based on neurovascular coupling, which measures cortical hemodynamic responses in real time [10, 11]. Although fNIRS has a lower spatial resolution than PET, it offers advantages including ease of use, high temporal resolution, and compatibility with electrophysiological monitoring [12]. Therefore, it is increasingly applied in clinical neuroscience research. The verbal fluency task (VFT), which engages frontal-temporal-parietal networks, is widely used to assess cognitive functions including language and executive control [13, 14], and has demonstrated sensitivity to cognitive impairment and compensatory mechanisms in epilepsy [15, 16]. The rationale of using task-based fNIRS hypoactivation as a potential indicator of EZ is based on the well-established phenomenon of interictal functional impairment in epilepsy. Chronic recurrent seizures can lead to reduced neuronal density, synaptic dysfunction, and altered metabolic baseline in regions involved in seizure generation and propagation. These functional deficits are not limited to the resting state; they also manifest as reduced task-evoked hemodynamic responses in cortical regions that are either part of the epileptogenic network or functionally connected to it. However, it must be emphasized that the task-related hypoactivation is not specific to the epileptogenic network, as it can also be attributed to variable task performances, antiseizure medication effects, preoperative cognitive deficits and generalized network dysfunction. In the present study, fNIRS-VFT was used as an exploratory tool and its interpretation should be integrated with multimodal data.

Notably, PET and fNIRS detect distinct yet physiologically coupled neural signals. PET measures static resting-state cerebral glucose metabolism reflecting the baseline neuronal activity, whereas fNIRS captures dynamic task-triggered hemodynamic changes that represent real-time cortical functional activation. The two modalities therefore provide complementary metabolic and functional information for multimodal presurgical evaluation.

Currently, the utility of fNIRS for EZ localization remains unclear, and postoperative verbal memory decline is a growing concern in epilepsy surgery. Although related to different clinical endpoints, localization accuracy versus cognitive preservation, the two questions are mechanistically linked: epileptogenic network pathology reduces both task-evoked activation (useful for lateralization/localization) and functional connectivity (reflecting network reserve and compensatory potential). Addressing the two questions in the same cohort enables a preliminary evaluation of fNIRS as both an adjunct to PET for EZ localization and an exploratory predictor of memory outcomes. The present study therefore had two primary objectives: (1) to compare the effectiveness of ^18^F-FDG-PET and fNIRS-VFT in presurgical localization of the EZ, and evaluate whether fNIRS-VFT provides complementary information when PET is inconclusive; and (2) to investigate whether task-based fNIRS functional connectivity during the verbal fluency task is associated with postoperative verbal memory decline.

## Materials and methods

### Patients

Patients with temporal or frontal epilepsy who underwent presurgical evaluation at our center between December 2022 and December 2023 were retrospectively employed. All patients received vEEG monitoring, high-resolution MRI, and ^18^F-FDG-PET for lateralization and localization. When noninvasive evaluations were inconclusive, sEEG was performed. For seizure-free patients, concordance with the surgical region was used as a pragmatic postsurgical reference for localization. Exclusion criteria included: (1) non-focal epilepsy; (2) inability to complete verbal memory assessment; and (3) severe neuropsychiatric comorbidity. This study was approved by the Ethics Committee of the First Affiliated Hospital of Fujian Medical University (No. 2024 − 514).

### ^18^F-FDG-PET/MRI

PET/MRI examinations were performed on a SIGNA PET/MR scanner (GE Healthcare, Milwaukee, WI). All patients were fasted for at least 6 h and were scanned only when blood glucose levels were < 200 mg/dL. A dose of ^18^F-FDG (3.7 MBq/kg) was administered intravenously, and PET acquisition consisted of a 20-min 3D brain scan obtained approximately 60 min after injection. MRI was acquired simultaneously using a standardized protocol, including 3D T1-weighted BRAVO, T2-weighted, T2-FLAIR, diffusion-weighted (b = 0 and 1000 s/mm²), and 3D arterial spin labeling sequences. All scans were performed during the interictal period. Images were assessed by two experienced nuclear medicine physicians blinded to all clinical, EEG, MRI (during initial reading), and surgical outcome data; MRI was available for anatomical correlation after the independent PET reading. Disagreements were resolved by consensus, or by a third senior reader if needed; inter-rater agreement was assessed (Cohen’s kappa). Evaluations were made visually, without semi-quantitative analysis. Based on visual observation, reduced uptake in comparison to the contralateral homotopic area was defined as hypometabolism. Concordance with the surgical EZ was defined at two levels: (1) lateralization—hypometabolism in the same hemisphere; and (2) localization—hypometabolism involved the resected/disconnected lobar region, irrespective of extension to other lobes. All results were re-confirmed before final data entry.

### fNIRS recording

**Fig. 1:**
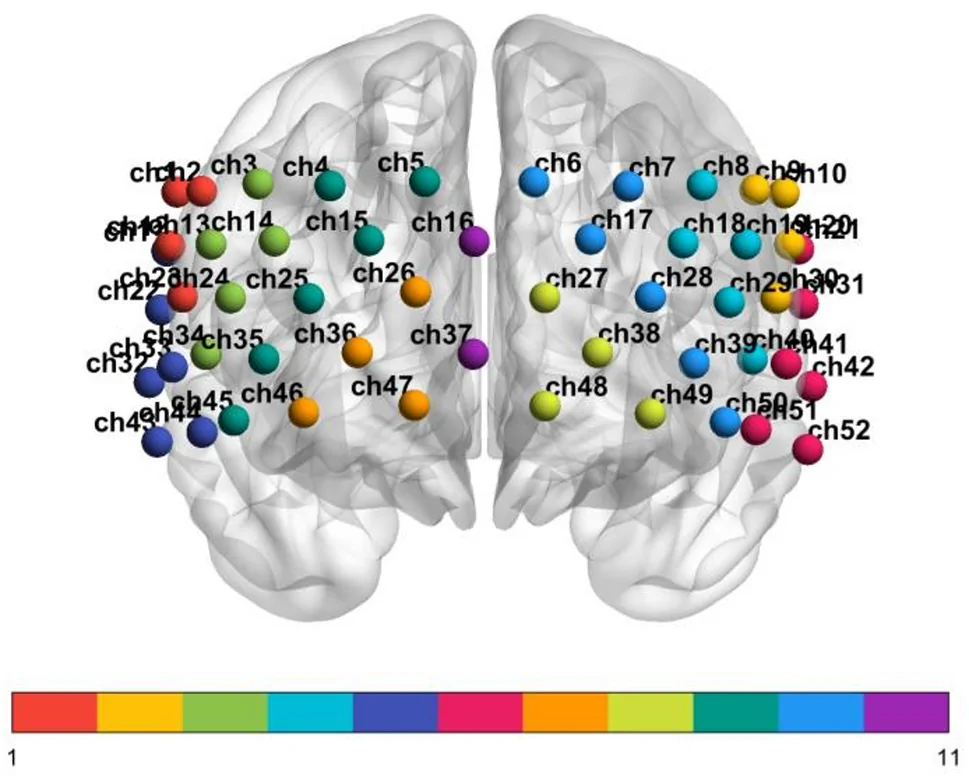
Cortical projection of the 52-channel NIRS. Adapt from Tung et al. [16]

**Fig. 2:**
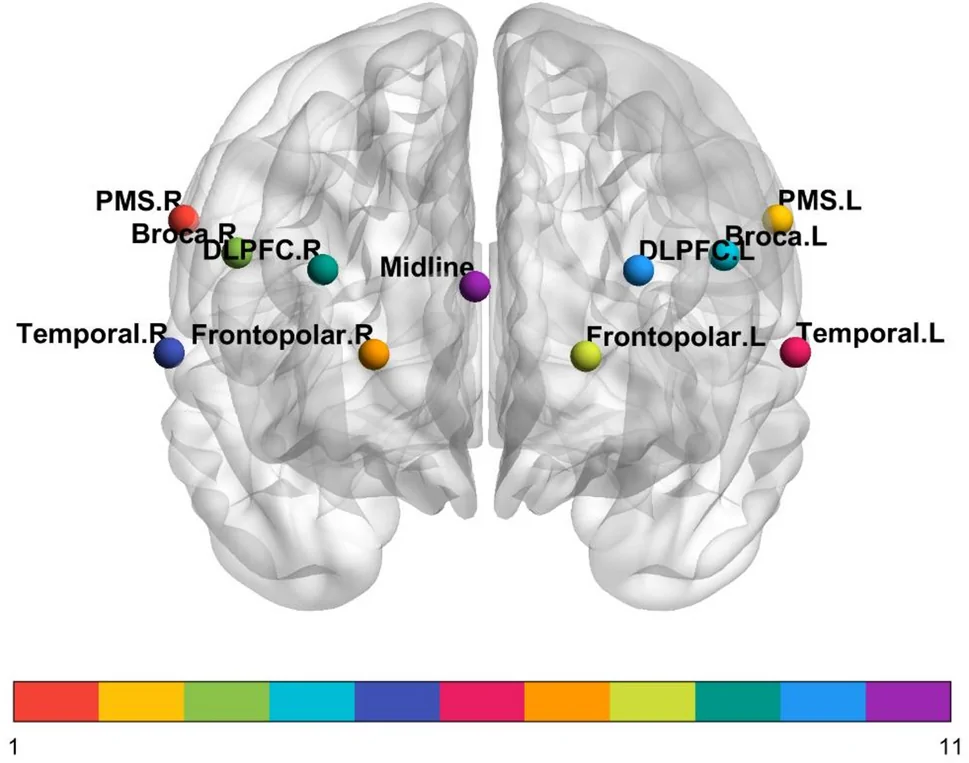
Mapping of the 52-channel NIRS on 11 anatomical brain regions. The 11 brain regions were region 1, Right Premotor, Motor, and Somatosensory Cortex (PMS.L, covered by channels 1, 2, 12, and 23); region 2, Left Premotor, Motor, and Somatosensory Cortex (PMS.R, covered by channels 9, 10, 20, and 30); region 3, Broca.R, covered by channels 3, 13, 14, 24, and 34; region 4, Broca.L, covered by channels 8, 18, 19, 29, and 40; region 5, Temporal.R, covered by channels 11, 22, 32, 33, 43, and 44; region 6, Temporal.L, covered by channels 21, 31, 41, 42, 51, and 52; region 7, Frontopolar.R, covered by channels 26, 36, 46, and 47; region 8, Frontopolar.L, covered by channels 27, 38, 48, and 49; region 9, Right Dorsolateral Prefrontal Cortex (DLPFC.R, covered by channels 4, 5, 15, 25, 35, and 45); region 10, Left Dorsolateral Prefrontal Cortex (DLPFC.L, covered by channels 6, 7, 17, 28, 39, and 50); and region 11, Midline, covered by channels 16 and 37. Adapt from Tung et al. [16]

fNIRS data were collected preoperatively and at ≥ 1 month postoperatively. This time point was selected for two reasons: (1) it avoids the acute post-surgical period (first 2 weeks) in which cortical edema and perioperative hemodynamic changes may affect fNIRS signals; and (2) it allows us to capture early postoperative changes related to resection rather than compensatory plasticity, as long-term functional reorganization may occur beyond 3 months post-surgery. All patients had uncomplicated postoperative recovery with no evidence of surgical complications before fNIRS acquisition. The fNIRS recording used a 52-channel system (ETG-4100, Hitachi) with 3 × 11 optode arrays covering the frontal and anterior temporal areas according to the international 10–20 system. Dual wavelengths (695 and 830 nm) were used, and data were sampled at 10 Hz. Channels were grouped into predefined cortical regions for analysis, based on the same parcellation scheme as described by Tung et al. [16]. (Figures 1 and 2).

### VFT

Participants performed a phonemic VFT during fNIRS recording. The recording consisted of a 30-s pre-task baseline, a 60-s task period (three 20-s blocks), and a 70-s post-task baseline. During the task blocks, participants were required to generate as many words as possible, beginning with a given Chinese phoneme. A brief practice session was provided to ensure task comprehension. The detailed task process is provided in the supplementary materials.

### fNIRS data processing

Oxygenated hemoglobin (HbO) signals were analzyed in MATLAB (R2022b).

#### Activation analysis

Data preprocessing and general linear model (GLM) analysis were performed using NIRS-SPM. After motion correction and temporal filtering, β-values reflecting task-related activation were computed for each channel and averaged within each region of interest (ROI).

#### Functional connectivity (FC) analysis

FC was estimated using the Homer2 toolbox. HbO time series from each ROI were averaged. Pearson correlation coefficients between ROI pairs were computed and Fisher z-transformed for statistical analysis. Detailed preprocessing algorithms, motion-correction procedures, and channel-pruning criteria are provided in Supplementary Materials.

### Verbal memory assessment

Verbal memory was assessed preoperatively and at ≥ 1 month postoperation (simultaneously with fNIRS-VFT) using the Chinese version of the Auditory Verbal Learning Test (AVLT). The test has been validated in Chinese-speaking populations with epilepsy [17], and focuses on delayed recall. Clinically significant decline was defined as a ≥ 4-point reduction, based on the Reliable Change Index [18, 19]. To exclude the influence of language deficits, language function was assessed using the Chinese version of the Western Aphasia Battery Aphasia Quotient (WAB-AQ), with ≥ 5-point change considered clinically meaningful [20]. Both instruments have demonstrated good reliability and validity in Mandarin-speaking clinical populations.

### Surgery and follow-up

All patients underwent ablative epilepsy surgery by the same senior surgeon. Surgical intervention area was defined as the cortical region lesioned during epilepsy surgery, as confirmed by postoperative MRI performed within 1 month after surgery. For each patient, postoperative T1-weighted MRI was coregistered with preoperative PET and with the fNIRS channel space using Statistical Parametric Mapping (SPM12) and the NIRS-SPM toolbox, respectively. Coregistration was performed to determine whether preoperative PET hypometabolism or fNIRS-VFT hypoactivation spatially overlapped with the surgical area. Concordance was classified as: [1] fully concordant (area of hypometabolism or hypoactivation completely within the surgical area); [2] localizing (correct identification of the specific lobe, and the area of abnormality included the surgical area but also extended beyond it); [3] lateralizing (correct identification of the affected hemisphere); and [4] negative (normal PET/fNIRS findings or the PET/fNIRS abnormalities were discordant with the surgical area). Postoperative seizure outcomes were evaluated at 2 years after surgery using Engel and ILAE classifications. Engel I/ILAE 1–2 outcomes were considered accurate localization.

### Data collection

Demographic and clinical variables were recorded, including age, sex, age at seizure onset, disease duration, PET findings, fNIRS activation patterns, and AVLT results. PET-positive (PET^+^) indicates hypometabolism within the surgical intervention area. fNIRS-positive (fNIRS+) indicates β < 0 within the surgical intervention area which suggests reduced activation). Memory decline was defined as ≥ 4-point decrease in AVLT delayed recall score.

## Results

### Clinical characteristics

Twenty-eight patients with DRE were included (11 males, 17 females), with mean age at seizure onset of 15.6 ± 6.3 years, age at surgery of 23.5 ± 7.3 years, and disease duration of 7.9 ± 5.5 years (Tables 1 and 2). At 2-year follow-up, 20 patients achieved Engel I/ILAE 1–2 outcomes.

**Table 1:** Demographic characteristics of participants

Patients	Sex	Age at seizure onset (years)	Disease duration (years)	Age at surgery (years)	Surgical outcome (Engel)
Sub01	Female	23	10	33	I
Sub02	Female	18	5	23	III
Sub03	Female	12	4	16	I
Sub04	Female	23	3	26	I
Sub05	Female	10	21	31	I
Sub06	Male	19	1	20	I
Sub07	Male	10	10	20	I
Sub08	Male	18	7	25	I
Sub09	Male	26	3	29	II
Sub10	Female	20	4	24	I
Sub11	Female	20	10	30	III
Sub12	Male	22	10	32	I
Sub13	Male	10	3	13	I
Sub14	Female	12	19	31	I
Sub15	Female	9	8	17	II
Sub16	Female	8	12	20	I
Sub17	Male	9	9	18	III
Sub18	Male	14	5	19	I
Sub19	Male	14	20	34	I
Sub20	Female	25	2	27	III
Sub21	Female	10	2	12	I
Sub22	Male	13	11	24	I
Sub23	Female	23	10	33	II
Sub24	Female	10	1	11	II
Sub25	Female	3	10	13	I
Sub26	Male	22	10	32	I
Sub27	Female	22	7	29	I
Sub28	Female	12	3	15	I

**Table 2:** Clinical characteristics of participants

Participants (*N* = 28)		
Sex, female, *n* (%)		17(60.7)
Age at epilepsy onset, years, mean (SD)		15.6 (6.3)
Age at surgery, years, mean (SD)		23.5 (7.3)
Duration of epilepsy, years, mean (SD)		7.9 (5.5)
Side of seizure, left, *n* (%)		19 (67.9)
MRI finding, positive, *n* (%)		8 (28.6)
Surgical region, *n* (%)		
Temporal		13 (46.4)
Frontal		15 (53.6)
Surgical types, *n* (%)		
Resection		5 (17.9)
LITT		16 (57.1)
RF-TC		7 (25)

### Localization performance of PET and fNIRS-VFT

#### ^18^F-FDG PET/MRI

Among the 20 seizure-free patients, 14 (70%) were PET^+^, showing interictal hypometabolism involving the surgical intervention area. Of them, hypometabolism was fully concordant with the resection area in 5 cases, localizing in 6 cases, and lateralizing only in 3 cases. Six patients (30%) showed no relevant hypometabolism (PET^−^) (Tables 3 and 4).

**Table 3:** PET and fNIRS-VFT results in Engel I patients

PET finding (*N* = 20)		fNIRS-VFT activation findings (*N* = 20)	
PET^+^	14 (70%)	fNIRS^+^	9 (45%)
fully concordant	5	fully concordant	0
localizing	6	localizing	4
lateralizing	3	lateralizing	5
PET^-^	6 (30%)	fNIRS^-^	11 (55%)
PET^+^/fNIRS^+^		8	
PET^+^/fNIRS^-^		6	
PET^-^/fNIRS^+^		1	
PET^-^/fNIRS^-^		5

**Table 4:** PET and fNIRS-VFT results in Engel I patients (patient-level)

Patients	fNIRS	PET
sub01	fully concordant	localizing
sub03	localizing	negative
sub04	negative	negative
sub05	localizing	lateralizing
sub06	fully concordant	localizing
sub07	fully concordant	negative
sub08	negative	negative
sub10	negative	negative
sub12	lateralizing	localizing
sub13	negative	lateralizing
sub14	lateralizing	localizing
sub17	fully concordant	lateralizing
sub18	fully concordant	lateralizing
sub19	negative	negative
sub21	localizing	negative
sub22	localizing	negative
sub25	localizing	negative
sub26	localizing	negative
sub27	negative	negative
sub28	lateralizing	lateralizing

#### fNIRS-VFT activation

**Fig. 3:**
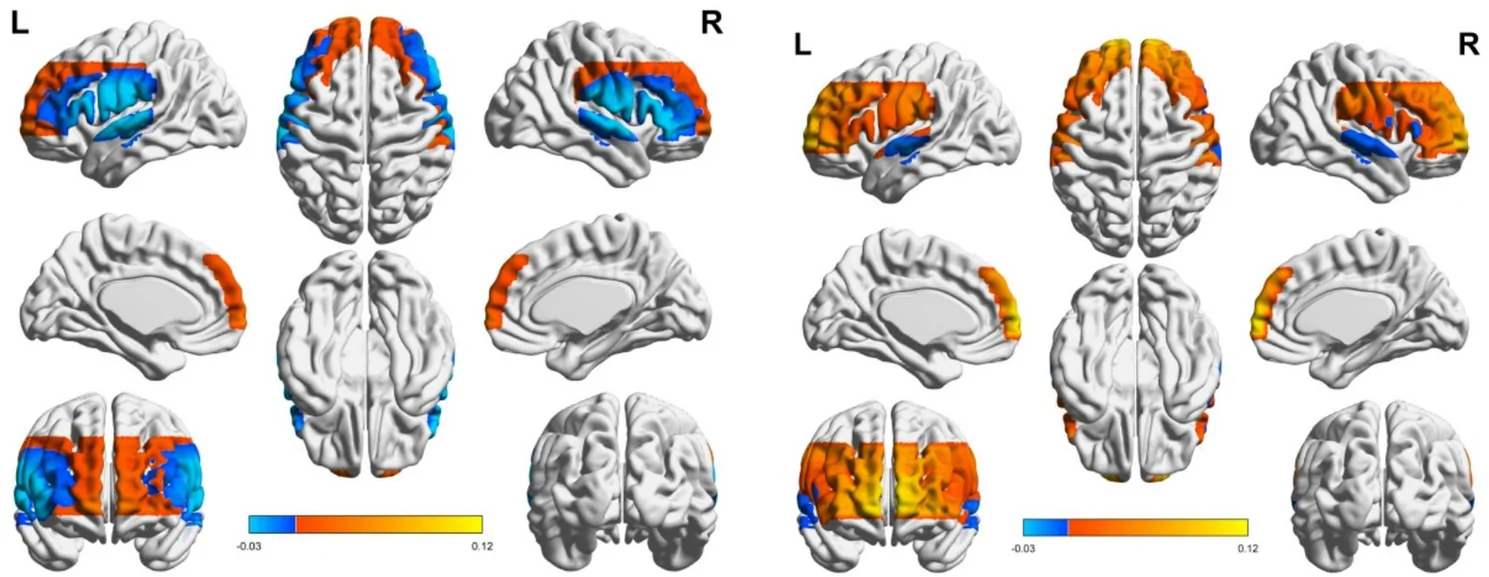
Hemodynamic responses in the anterior frontal and temporal lobes measured by fNIRS-VFT in two patients. Regions with β < 0 are colored blue, while regions with β > 0 are colored red. The positive and negative β values merely denote differential activation patterns during the verbal fluency task

Among the 20 seizure-free patients, 9 (45%) exhibited fNIRS-VFT hypoactivation involving the surgical intervention area (fNIRS^+^), including 4 cases showing localizing patterns and 5 showing lateralizing only. No patient had hypoactivation fully concordant with the surgical site. The remaining 11 patients (55%) showed no relevant hypoactivation (fNIRS^−^) (Tables 3 and 4; example shown in Fig. 3).

### Postoperative verbal memory and fNIRS functional connectivity

#### AVLT outcomes

Nineteen patients completed both pre- and postoperative AVLT. Four showed ≥ 4-point decline in delayed recall, while 15 had stable performance. No patient demonstrated a ≥ 5-point reduction in WAB-AQ, excluding language impairment as a cause of VFT performance differences (Table 5).

#### fNIRS functional connectivity analysis

**Fig. 4:**
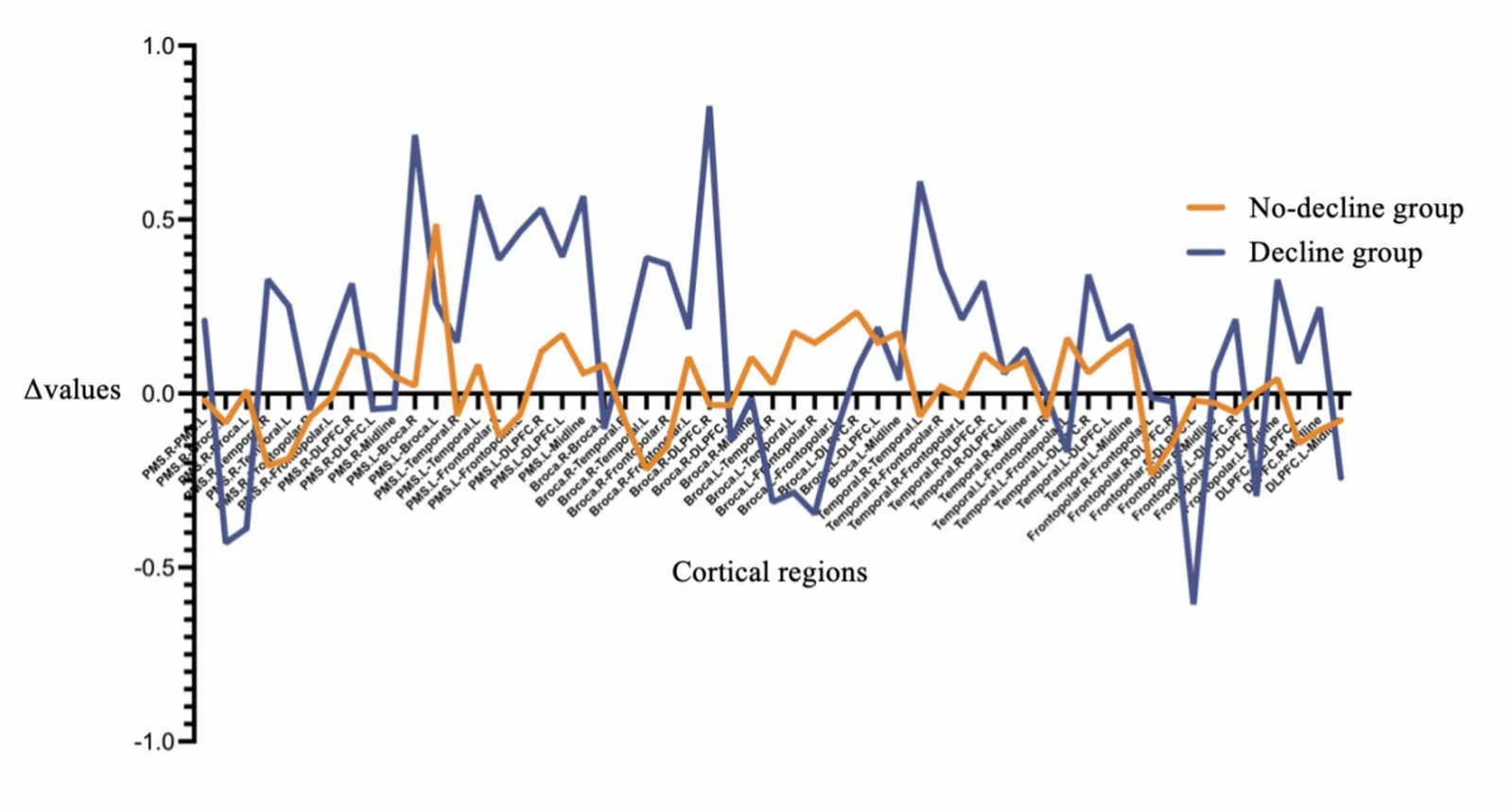
Functional connectivity Δ values (postoperative minus preoperative) in patients with and without postoperative verbal memory decline

**Fig. 5:**
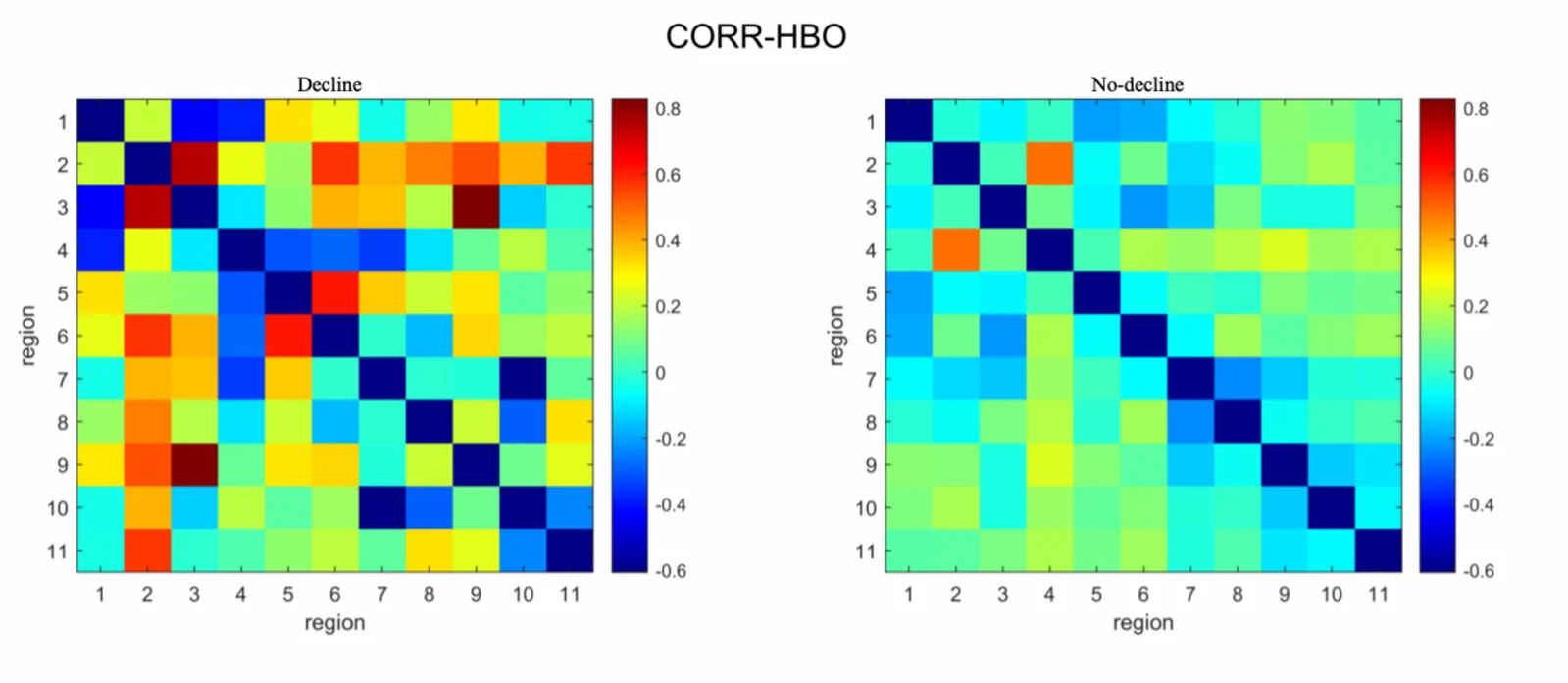
Mean Δvalues matrix indicating functional connectivity among the 11 anatomical brain regions in patients with and without postoperative verbal memory decline

Nineteen patients were analyzed, including 4 with postoperative memory decline and 15 without. The memory decline group showed larger and more polarized connectivity changes, particularly in Frontopolar. R–DLPFC.L, PMS.R–Broca.L, and PMS.R–Broca.R connectivities. Several connections in the memory decline group exhibited opposite-direction changes (e.g., PMS.R–Temporal.R, PMS.R–Temporal.L) and pronounced disinhibition (Frontopolar.R–DLPFC.L) compared with non-decliners (Table 6; Figs. 4 and 5).

**Table 5:** Preoperative and postoperative cognitive scores of individuals

Patients	MoCA- pre	MoCA- post	WAB- pre	WAB- post	WAB- AQ	AVLT immediately-pre	AVLT immediately-post	AVLT delay-pre	AVLT delay-post	Δvalues*
Sub02	15	16	96.3	96.2	-0.1	32	46	7	6	-1
Sub03	19	22	96.9	97.7	0.8	39	33	7	4	-3
Sub04	28	30	98.5	98.8	0.3	57	47	11	7	-4
Sub07	25	26	97.5	97.9	0.4	32	31	3	4	1
Sub09	25	23	98.3	97.7	-0.6	43	28	6	2	-4
Sub11	29	27	99.6	98.2	-1.4	49	42	10	12	2
Sub12	15	27	94.5	99.4	4.9	12	39	0	10	10
Sub13	30	30	98.8	99.2	0.4	47	41	10	7	-3
Sub14	28	27	98.4	97	-1.4	52	49	11	11	0
Sub15	26	26	98.5	99.5	1	52	52	14	8	-6
Sub18	23	27	100	99.6	-0.4	62	64	10	10	0
Sub19	19	19	94	90.6	-3.4	34	30	6	0	-6
Sub20	27	24	97	96.5	-0.5	38	48	9	9	0
Sub21	27	25	99.6	98.7	-0.9	37	52	8	11	3
Sub22	26	28	97.5	98.5	1	44	36	10	9	-1
Sub25	22	24	93.6	95.3	1.7	39	45	4	9	5
Sub26	20	24	95.4	96.9	1.5	30	28	1	2	1
Sub27	28	25	98.2	96.8	-1.4	46	74	3	14	11
Sub28	17	19	97.3	95.6	-1.7	29	30	9	6	-3

* Δvalues = AVLT delay-post - AVLT delay-pre

**Table 6:** Exploratory descriptive comparison of the top 10 functional connections with Δvalue differences between groups

Cortical regions	Δ values in patient without postoperative verbal memory decline	Δ values in patients with postoperative verbal memory decline	Difference in Δ values between groups
Frontopolar.R–DLPFC.L	-0.02	-0.606	0.586
PMS.R–Broca.L	0.009	-0.387	0.396
PMS.R–Broca.R	-0.085	-0.429	0.344
Frontopolar.L–DLPFC.L	0.002	-0.295	0.297
Frontopolar.R–Midline	-0.028	0.061	-0.089
PMS.R–DLPFC.R	0.123	0.316	-0.193
Frontopolar.L–DLPFC.R	-0.055	0.213	-0.268
DLPFC.R–Midline	-0.105	0.247	-0.352
PMS.R–Temporal.L	-0.187	0.251	-0.438
PMS.R–Temporal.R	-0.208	0.328	-0.536

Δ value: postoperative minus preoperative functional connectivity

## Discussion

This study had two primary objectives: to compare the EZ localization performances of F-FDG-PET and task-based fNIRS-VFT in patients with DRE, and to explore associations between task-evoked fNIRS functional connectivity and postoperative verbal memory decline. Our key findings are as follows. First, PET showed higher concordance with surgical areas than fNIRS-VFT in the seizure-free patients, while fNIRS provided complementary lateralizing information in one case with non-localizing PET results. Second, the task-based fNIRS connectivity changes may be correlated with postoperative verbal memory alterations. All interpretations herein are hypothesis-generating rather than definitive causal conclusions.

### Localization performance of PET and fNIRS-VFT

#### PET and EZ localization

^18^F-FDG-PET exhibited higher concordance with surgical areas compared to fNIRS-VFT among seizure-free DRE patients. In cases with favorable postoperative outcomes, PET achieved a 70% positivity rate and precise EZ localization in 55% of patients, slightly lower than the accuracy of high-resolution MRI [21, 22]. Consistent with prior work, interictal PET hypometabolism frequently extended beyond the resected EZ, with 64.3% of PET-positive patients showing hypometabolic areas larger than the surgical region. Such widespread hypometabolism reflects diffuse epileptic network dysfunction driven by neuronal loss, altered synaptic activity, chronic seizure burden, and antiseizure medication effects [23–25]. Accordingly, PET hypometabolism covers not only the focal EZ but also surrounding dysfunctional cortical networks [26]. Complementary graph-based PET analyses further support its utility in presurgical evaluation by characterizing metabolic network disturbances and predicting surgical outcomes [27, 28].

#### fNIRS-VFT and EZ localization

Unlike PET, fNIRS measures cortical hemodynamics and thus reflects functional metabolic activity rather than resting glucose utilization. The present fNIRS-VFT paradigm yielded a 45% positivity rate for EZ localization, indicating limited clinical utility when used alone. Nevertheless, this rate exceeds the previously reported rate for resting-state fNIRS detection (6–24%) [29], suggesting that the task-based paradigms may offer modest advantages for epileptic lateralization. Clinically, fNIRS-VFT correctly lateralized the EZ in one patient with non-lateralizing PET, indicating its potential use as a complementary tool for patients with ambiguous imaging findings. However, this single-case observation cannot be generalized and remains purely hypothesis-generating. Notably, this study lacked quantitative volumetric analysis to compare the spatial extent of fNIRS hypoactivation and PET hypometabolism. Any perceived differences in signal coverage were based on visual observation and were descriptive without statistical validation, and thus no definitive spatial inferences are drawn here. Though technically limited when used alone, fNIRS offers unique practical benefits including free from radiation, high temporal resolution, portability, and naturalistic task testing. Combining fNIRS-EEG integration and novel metabolic biomarkers such as Cytochrome c oxidase may further improve the detection accuracy in future work [30–32]. Therefore, fNIRS is a supplementary presurgical modality rather than a replacement for PET.

### fNIRS functional connectivity and postoperative verbal memory

This exploratory analysis examined preliminary, uncorrected associations between task-based fNIRS connectivity patterns and postoperative verbal memory decline. Compared to the group with no memory decline, patients with memory impairment (*n* = 4) showed prominent, divergent frontotemporal network changes, including reduced connectivity in DLPFC, frontopolar, Broca’s, and premotor pathways. These group-level differences are purely descriptive, with no correction for multiple comparisons, limiting the interpretive robustness. Given the established roles of the DLPFC in working memory and attentional control and the role of frontopolar cortex in strategic memory retrieval [33–35], reduced connectivity within these circuits may hypothetically disrupt verbal memory processing. Conversely, increased premotor-temporal connectivity in the memory decline group may reflect inefficient compensatory network reorganization linked to hippocampal-dependent memory function [36]. Importantly, these mechanistic interpretations remain speculative. Due to the small sample size and the uncorrected analyses, the findings in this study could not confirm the causal links between connectivity dysregulation and memory decline, or distinguish between true adaptive reorganization and nonspecific postoperative network changes. However, the postoperative verbal memory decline appears to correlate with the widespread task-related network disturbances rather than focal cortical dysfunction. The task-based fNIRS connectivity may be a radiation-free marker for postoperative cognitive monitoring, which requires large-scale validation in future studies.

In summary, fNIRS plays a distinct, complementary role in multimodal epilepsy evaluation. As a presurgical tool, fNIRS-VFT is complementary to PET for EZ localization in PET-inconclusive cases. For postoperative monitoring, task-based fNIRS connectivity provides a repeatable, safe approach to characterizing memory-related network alterations. Our findings are preliminary and hypothesis-generating due to the small sample size and lack of quantitative spatial and statistical correction. Future multimodal integration of fNIRS with EEG or fMRI will help validate these observations and improve presurgical localization and cognitive outcome prediction.

## Limitations

Despite these promising findings, some limitations in this study should be noted. First, this retrospective single-center study was subject to selection bias and reduced statistical generalizability due to the small sample size and limited memory-decline subgroup. Second, the restricted fNIRS coverage on frontal and anterior temporal areas excluded deep structures related with memory, such as the hippocampus, while the absence of short-channel correction may leave extracerebral artifacts uncorrected. Third, the interindividual variability in antiseizure medications may further confound the hemodynamic and connectivity results. Notably, functional connectivity was estimated without removing the task-evoked response, and the resulting correlations may partly reflect shared task timing rather than pure interregional neural coupling. In addition, the lack of healthy and non-surgical epilepsy control groups prevented differentiation of surgery-associated network changes from inherent epileptic abnormalities. Without systematic behavioral VFT analysis and formal language dominance evaluation, the functional and cognitive interpretations of the task-evoked connectivity alterations remain constrained. Accordingly, our findings are exploratory and hypothesis-generating. Future prospective multicenter studies with larger cohorts, controlled grouping, standardized fNIRS artifact correction, behavioral and language assessment, and medication stratification are required to validate the clinical value of task-based fNIRS in presurgical epilepsy evaluation and postoperative cognitive monitoring.

## Conclusions

This exploratory retrospective study provides preliminary evidence for two potential applications of fNIRS-VFT in the surgical management of drug-resistant epilepsy. First, although interictal ^18^F-FDG PET showed higher concordance with the surgically treated region than fNIRS-VFT in seizure-free patients, fNIRS-VFT provided potentially useful lateralizing information in one PET-nonlocalizing case, suggesting that fNIRS-VFT is worth further investigation as an adjunctive tool. Second, postoperative verbal memory decline may be accompanied by changes in task-related fNIRS connectivity, but these findings were from a very small subgroup and require validation in prospective studies.

## Supplementary Information

Below is the link to the electronic supplementary material.

Supplementary Material 1

## Data Availability

The data that support the findings of this study are available from the corresponding author upon reasonable request.

## Abbreviations

AVLT: Auditory verbal learning test
DLPFC: Dorsolateral prefrontal cortex
DRE: Drug-resistant epilepsy
EEG: Electroencephalography
EZ: Epileptogenic zone
FC: Functional connectivity
FDG: Fluorodeoxyglucose
fMRI: Functional magnetic resonance imaging
fNIRS: Functional near-infrared spectroscopy
fNIRS-VFT: Functional near-infrared spectroscopy during a verbal fluency task
GLM: General linear model
HbO: Oxygenated hemoglobin
ILAE: International League Against Epilepsy
MRI: Magnetic resonance imaging
PET: Positron emission tomography
PMS: Premotor, motor, and somatosensory cortex
ROI: Region of interest
sEEG: stereoelectroencephalography
SPM: Statistical parametric mapping
VFT: Verbal fluency task
vEEG: video electroencephalography
WAB-AQ: Western Aphasia Battery aphasia quotient
