# Supplementary material for: fNIRS-VFT is a complementary tool to 18F-FDG PET in presurgical evaluation of drug-resistant epilepsy: an exploratory study
Source: Acta Epileptol. 2026 Oct 26;8:37. doi: 10.1186/s42494-026-00282-2 (PMC13711974; doi:10.1186/s42494-026-00282-2)
Supplement: Supplementary file 1 [file 42494_2026_282_MOESM1_ESM.docx]

**S1. Verbal Fluency Task (VFT) Procedure**

Each participant completed the VFT during fNIRS recording.

The task consisted of three phases:

①Pre-task baseline (30 s): Count repeatedly from 1–5

②Task period (60 s): Three consecutive 20-s blocks without inter-block intervals

③Post-task baseline (70 s): Repeat counting from 1–5

The baseline condition consisted of repeated counting from 1 to 5, which was chosen as an active control for basic articulatory‑motor output and sustained attention, rather than a neutral resting state. We acknowledge that this is not a true resting baseline; consequently, this approach may reduce the contrast‑to‑noise ratio and potentially underestimate task‑related activation. We consider this a conservative strategy that minimizes false‑positive findings.

During task blocks, participants generated as many words as possible beginning with a given Chinese phoneme (e.g., “bai”).

Participants received standardized instructions and completed one practice session prior to testing.

**S2. fNIRS Data Preprocessing and Activation Analysis**

**S2.1 Data Conversion**

Raw CSV files were converted to MATLAB .mat format for NIRS-SPM processing.

**S2.2 Preprocessing Pipeline (NIRS-SPM)**

**All preprocessing steps were applied to the entire continuous recording (including pre‑task baseline, task period, and post‑task baseline) before any segment extraction. The sequential steps were as follows:**

①Wavelet-MDL detrending to remove baseline drift caused by respiration, heartbeat, vascular pulsation, and instrumental instability

②Bad Channel Detection using enPruneChannels: A channel was marked as bad if: Mean light intensity <1 or >10; Signal-to-noise ratio (SNR) <2.

Additional manual inspection was performed to identify artifact-contaminated channels or time windows.

③Motion artifact detection and correction: artifacts were detected using hmrMotionArtifactByChannel (threshold: amplitude change >5 within 2 s or >50× SD; ±4‑s window around detected artifacts flagged) and corrected using cubic spline interpolation (hmrMotionCorrectSpline, p = 0.99).

④Band‑pass filtering using hmrBandpassFilt: high‑pass cutoff 0.01 Hz, low‑pass cutoff 0.1 Hz (third‑order Butterworth). Filtering was applied to the full‑length recording to avoid edge artifacts and ensure adequate data length for the low‑frequency filter.

**S2.3 GLM Model Construction**

General linear model (GLM) estimation: HbO concentration time series were modeled using the canonical hemodynamic response function (HRF) with temporal and dispersion derivatives as basis functions. The design matrix included the VFT block timing as the independent variable; HbO concentration as the dependent variable. β‑values were extracted per channel.

ROI‑level activation: mean β‑value across valid channels within each ROI was computed. Design matrices were generated for each subject for quality inspection (see Figure S1).

**S3. Functional Connectivity Analysis**

Functional connectivity (FC) analysis was conducted using the Homer2 toolbox. All preprocessing steps described below were applied to the entire continuous recording prior to segment extraction.

**S3.1 Data Segment Extraction**

The 30–90 s interval of the task period (total 60 s) was extracted for FC calculation.

**S3.2 Bad Channel Detection**

Using enPruneChannels:

A channel was marked as bad if:

①Mean light intensity <1 or >10

②Signal-to-noise ratio (SNR) <2

Additional manual inspection was performed to identify artifact-contaminated channels or time windows.

**S3.3 Motion Artifact Detection and Correction**

①Detection: hmrMotionArtifactByChannel

·Threshold: amplitude change >5 within 2 s or >50× SD

·A±4 s window around detected artifacts was flagged

②Correction: cubic spline interpolation (hmrMotionCorrectSpline, p = 0.99)

**S3.4 Band-pass Filtering**

Applied using hmrBandpassFilt:

①High-pass cutoff: 0.01 Hz

②Low-pass cutoff: 0.1 Hz (third-order Butterworth)

Removes cardiac (~1.6–1.8 Hz), and respiratory (~0.2–0.3 Hz) artifacts.

**S3.5 Conversion to Hemoglobin Concentrations**

①Modified Beer-Lambert law

②Differential pathlength factor (DPF): [6 6] for HbO/HbR

**S3.6 ROI-level Time Series**

Channels within each ROI were averaged after excluding bad channels.

**S3.7 Functional Connectivity Computation**

①Metric: Pearson correlation coefficient (CORR) between ROI pairs

②Values transformed using Fisher’s z for statistical analysis

Figure S1 The design matrix for an individual patient.

Table S1 Functional connectivity Δvalues in both groups

|  | Decline | No-decline |
| --- | --- | --- |
| PMS.R-PMS.L | 0.209526598 | -0.019493965 |
| PMS.R-Broca.R | -0.429202971 | -0.085357698 |
| PMS.R-Broca.L | -0.386900942 | 0.009153247 |
| PMS.R-Temporal.R | 0.328151055 | -0.207741038 |
| PMS.R-Temporal.L | 0.251276131 | -0.186834285 |
| PMS.R-Frontopolar.R | -0.046290869 | -0.06957051 |
| PMS.R-Frontopolar.L | 0.148256624 | -0.0135618 |
| PMS.R-DLPFC.R | 0.315940004 | 0.122755423 |
| PMS.R-DLPFC.L | -0.045224397 | 0.107389342 |
| PMS.R-Midline | -0.040803628 | 0.049523453 |
| PMS.L-Broca.R | 0.743143038 | 0.022558785 |
| PMS.L-Broca.L | 0.259585231 | 0.487121531 |
| PMS.L-Temporal.R | 0.147408893 | -0.064013266 |
| PMS.L-Temporal.L | 0.569370363 | 0.082573229 |
| PMS.L-Frontopolar.R | 0.385427941 | -0.123575976 |
| PMS.L-Frontopolar.L | 0.465796666 | -0.060770279 |
| PMS.L-DLPFC.R | 0.530944081 | 0.120462367 |
| PMS.L-DLPFC.L | 0.393361906 | 0.169670379 |
| PMS.L-Midline | 0.567141143 | 0.05807102 |
| Broca.R-Broca.L | -0.101148124 | 0.083287547 |
| Broca.R-Temporal.R | 0.13232339 | -0.082328148 |
| Broca.R-Temporal.L | 0.390281072 | -0.218322402 |
| Broca.R-Frontopolar.R | 0.370955059 | -0.150905256 |
| Broca.R-Frontopolar.L | 0.185557475 | 0.10425578 |
| Broca.R-DLPFC.R | 0.826638031 | -0.032711486 |
| Broca.R-DLPFC.L | -0.136186703 | -0.033848343 |
| Broca.R-Midline | -0.012556008 | 0.103951143 |
| Broca.L-Temporal.R | -0.311710213 | 0.0279334 |
| Broca.L-Temporal.L | -0.284479516 | 0.176786918 |
| Broca.L-Frontopolar.R | -0.345537661 | 0.14525059 |
| Broca.L-Frontopolar.L | -0.11045688 | 0.187662905 |
| Broca.L-DLPFC.R | 0.071339797 | 0.233846459 |
| Broca.L-DLPFC.L | 0.189250938 | 0.146000074 |
| Broca.L-Midline | 0.03940393 | 0.173893099 |
| Temporal.R-Temporal.L | 0.609570255 | -0.066013298 |
| Temporal.R-Frontopolar.R | 0.356548509 | 0.020055733 |
| Temporal.R-Frontopolar.L | 0.213373352 | -0.009711593 |
| Temporal.R-DLPFC.R | 0.322078239 | 0.114158693 |
| Temporal.R-DLPFC.L | 0.056674142 | 0.065641987 |
| Temporal.R-Midline | 0.129074858 | 0.091697654 |
| Temporal.L-Frontopolar.R | -0.002437313 | -0.070371298 |
| Temporal.L-Frontopolar.L | -0.167903846 | 0.159358823 |
| Temporal.L-DLPFC.R | 0.341665142 | 0.059357954 |
| Temporal.L-DLPFC.L | 0.154338612 | 0.110791502 |
| Temporal.L-Midline | 0.19579835 | 0.151969798 |
| Frontopolar.R-Frontopolar.L | -0.01184694 | -0.234232666 |
| Frontopolar.R-DLPFC.R | -0.022921559 | -0.143171916 |
| Frontopolar.R-DLPFC.L | -0.606396897 | -0.020057822 |
| Frontopolar.R-Midline | 0.060579699 | -0.027710832 |
| Frontopolar.L-DLPFC.R | 0.213286196 | -0.054571066 |
| Frontopolar.L-DLPFC.L | -0.294502103 | 0.001909627 |
| Frontopolar.L-Midline | 0.327269453 | 0.042442224 |
| DLPFC.R-DLPFC.L | 0.087589637 | -0.143127152 |
| DLPFC.R-Midline | 0.247483808 | -0.105221488 |
| DLPFC.L-Midline | -0.241798959 | -0.07711159 |
